# Identification and Validation of an Autophagy-Related lncRNA Signature for Patients With Breast Cancer

**DOI:** 10.3389/fonc.2020.597569

**Published:** 2021-02-05

**Authors:** Ruyue Zhang, Qingwen Zhu, Detao Yin, Zhe Yang, Jinxiu Guo, Jingmin Zhang, Yubing Zhou, Jane J. Yu

**Affiliations:** ^1^ Department of Oncology, The First Affiliated Hospital of Zhengzhou University, Zhengzhou, China; ^2^ Department of Pharmacy, The First Affiliated Hospital of Zhengzhou University, Zhengzhou, China; ^3^ Department of Otorhinolaryngology Head and Neck surgery, Children’s Hospital Affiliated to Zhengzhou University, Zhengzhou, China; ^4^ Department of Thyroid Surgery, The First Affiliated Hospital of Zhengzhou University, Zhengzhou, China; ^5^ Department of Internal Medicine, Pulmonary, Critical Care and Sleep Medicine, University of Cincinnati College of Medicine, Cincinnati, OH, United States

**Keywords:** long non-coding RNA, autophagy, breast cancer, The Cancer Genome Atlas, prognostic signature

## Abstract

**Background:**

Autophagy is a “self-feeding” phenomenon of cells, which is crucial in mammalian development. Long non-coding RNA (lncRNA) is a new regulatory factor for cell autophagy, which can regulate the process of autophagy to affect tumor progression. However, poor attention has been paid to the roles of autophagy-related lncRNAs in breast cancer.

**Objective:**

This study aimed to construct an autophagy-related lncRNA signature that can effectively predict the prognosis of breast cancer patients and explore the potential functions of these lncRNAs.

**Methods:**

The RNA sequencing (RNA-Seq) data of breast cancer patients was collected from The Cancer Genome Atlas (TCGA) database and the GSE20685 database. Multivariate Cox analysis was implemented to produce an autophagy-related lncRNA signature in the TCGA cohort. The signature was then validated in the GSE20685 cohort. The receiver operator characteristic (ROC) curve was performed to evaluate the predictive ability of the signature. Gene set enrichment analysis (GSEA) was used to explore the potential functions based on the signature. Finally, the study developed a nomogram and internal verification based on the autophagy-related lncRNAs.

**Results:**

A signature composed of 9 autophagy-related lncRNAs was determined as a prognostic model, and 1,109 breast cancer patients were divided into high-risk group and low-risk group based on median risk score of the signature. Further analysis demonstrated that the over survival (OS) of breast cancer patients in the high-risk group was poorer than that in the low-risk group based on the prognostic signature. The area under the curve (AUC) of ROC curve verified the sensitivity and specificity of this signature. Additionally, we confirmed the signature is an independent factor and found it may be correlated to the progression of breast cancer. GSEA showed gene sets were notably enriched in carcinogenic activation pathways and autophagy-related pathways. The qRT-PCR identified 5 lncRNAs with significantly differential expression in breast cancer cells based on the 9 lncRNAs of the prognostic model, and the results were consistent with the tissues.

**Conclusion:**

In summary, our signature has potential predictive value in the prognosis of breast cancer and these autophagy-related lncRNAs may play significant roles in the diagnosis and treatment of breast cancer.

## Introduction

Breast cancer is the most common malignant tumor and the leading cause of cancer-related deaths in women worldwide, with an incidence and mortality of 24.2% and 15.0%, respectively (1, 2). Current treatment options for breast cancer usually combine surgery with a variety of adjuvant therapies, such as chemotherapy, radiotherapy, endocrine therapy, targeted therapy, and immunotherapy (3–8). Most patients respond to initial treatment within a certain period of time, but there are still some breast cancers, especially triple negative breast cancer, that will develop into a more invasive tumor form, resulting in a poor prognosis (9–11). Because of the strong heterogeneity of breast cancer, multi-parameter signals are more valuable than single biomarkers in predicting the prognosis of breast cancer.

Autophagy is a physiological process that guides the degradation of damaged, denatured, or senescent proteins and damaged organelles in lysosomes. There are two states of autophagy activation: normal state and pathological state. Autophagy responds to various stresses by providing circulating metabolic substrates needed for survival under normal physiological conditions (12). Recent studies further indicate that abnormal autophagy is considered to be a potential cause of many diseases, including cardiovascular and cerebrovascular diseases, liver diseases, and cancer (13, 14). Autophagy is closely related to the occurrence, development, and metastasis of tumor. Multiple studies have reported that autophagy may play especially important roles in supporting cell growth and drug resistance to targeted therapy in BRAF-mutant melanoma (15–17). Some researchers found that MIR106A-5p upregulation suppressed autophagy and accelerated malignant phenotype in nasopharyngeal carcinoma (18). Furthermore, recent findings have revealed the autophagy drugs such as sirolimus and arsenic trioxide can induce autophagy of tumor cells and improve the outcome of certain cancer patients. However, only a limited proportion of patients benefited from autophagy-based treatment. Therefore, establishing predictive biomarkers for autophagic drug therapy response is required to identify patients who can benefit from autophagy-based treatment.

LncRNA is a small RNA with a length of more than 200bp and no protein coding function (19). Although they cannot be translated into proteins, they are involved in the process of protein translation (20). LncRNA plays a critical role in complex autophagy regulatory networks by regulating the biological effects of a variety of autophagy-related DNA, RNA, or proteins (21). Some studies have reported that lncRNA-HOTAIRM1 regulates autophagy and degrades tumor protein PML-RARA, during differentiation arrest of bone marrow cells, suggesting that lncRNA is a potential therapeutic target for leukemia (22). Other researchers have found the expression of lncRNA MEG3 is significantly down-regulated in glioma tissues and cells, and its overexpression can significantly inhibit cell proliferation and promote apoptosis and autophagy of glioma cells (23).

Considering the significance of lncRNA and autophagy in breast cancer biology, we determined an autophagy-related lncRNA signature to predict the prognosis of breast cancer patients and provide a theoretical basis for the diagnosis and treatment of breast cancer.

## Materials and Methods

### Datasets Preparation

**Figure 1:**
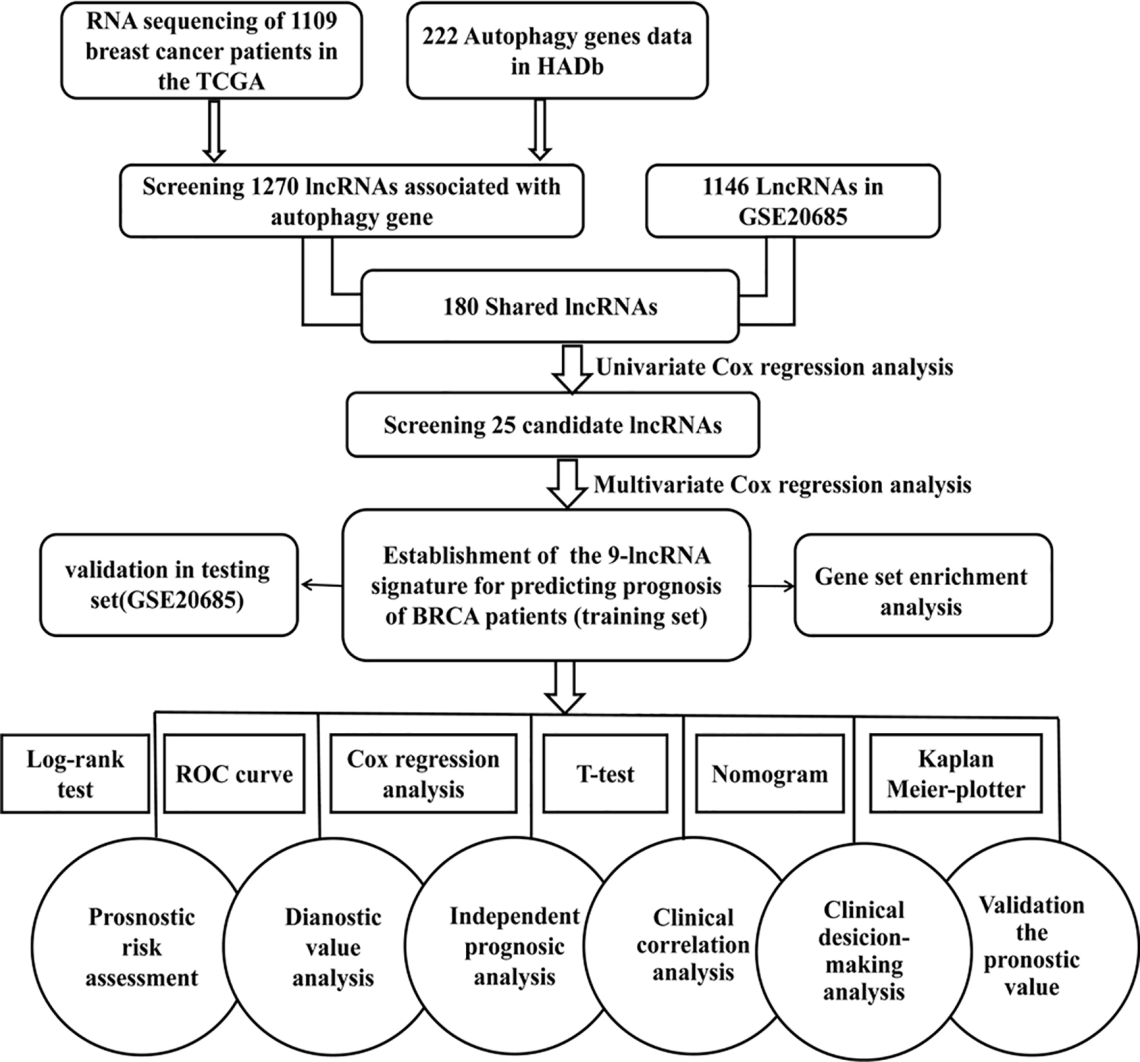
Flowchart for identification and validation of an autophagy-related lncRNA signature in breast cancer patients. The study was carried out in TCGA and GSE20685 database. The TCGA training set was used to identify prognostic lncRNAs. The multivariate Cox hazard model analysis was performed to construct a prognostic signature based on the prognostic lncRNAs. The prognosis signature was validated in the GSE20685 testing set.

The workflow of our analysis is illustrated in **Figure 1**. TCGA (https://cancergenome.nih.gov) RNA-Seq data of breast cancer patients was used as a training set to construct a signature composed of autophagy-related lncRNAs. The training cohort includes TGGA mRNA expression (FPKM) in 1,109 breast cancer patients and related clinical information. We collected an independent dataset GSE20685 (series matrix files), which was performed using the platform GPL570 [HG-U133_Plus_2] Affymetrix Human Genome U133 Plus 2.0 Array (https://www.ncbi.nlm.nih.gov/geo/query/acc.cgi?acc=GSE20685). In order to validate the predictive capability and the universal applicability of the signature, GSE20685 dataset including 327 breast cancer patients was employed as a testing set in our present study.

### LncRNAs and Autophagy Genes Mining

LncRNAs and mRNAs were extracted based on the annotations provided by GENCODE (https://www.gencodegenes.org). Obtain the list of autophagy gene from the human autophagy data (HADb, http://autophagy.lu/clustering/index.html) and extract the mRNA related to autophagy gene. Analyze the correlation between lncRNAs and autophagy-related genes in breast cancer by using Pearson method. LncRNAs with correlation coefficient R^2^ > 0.3 and *P* < 0.001 are considered to be autophagy-related lncRNAs. Extract the shared lncRNAs from autophagy-related lncRNAs and GSE20685 for the following analysis. Then, the shared lncRNAs in TCGA were used as a training dataset (n=1,109), and the shared lncRNAs in GSE20658 were used as a testing dataset (n=327). All RNA-sequencing data were normalized by log2 conversion.

### Signature Development

Univariate Cox regression analysis was executed to obtain the prognosis-related lncRNAs from the training dataset. Then the lncRNAs with *P* < 0.05 were included in the multivariate Cox hazard model analysis to produce an autophagy-related lncRNA signature model. In order to avoid over-fitting, Akaike Information Criterion (AIC) was conducted to select the stepwise signature, and the signature with the lowest AIC value was considered to be the optimal signature. The risk score of each patient with breast cancer was calculated according to the following formula: Risk score= β _lncRNA1_ × expr_lncRNA1_+ β_lncRNA2_ × expr_lncRNA2_+ …. + β_lncRNAn_ × expr_lncRNAn_. β is the regression coefficient of the corresponding lncRNA, expr is the expression level of lncRNA, and its unit is FPKM. Based on the median risk score, 1,109 patients with breast cancer in the training dataset were divided into two groups: high-risk group and low-risk group. In order to compare the difference of OS between high-risk group and low-risk group, Kaplan Meier-plotter was implemented. In addition, ROC curve was performed to evaluate the predictive ability of the signature by using the SurvivalROC package. Finally, 327 breast cancer patients with credible prognostic data from the GSE20685 set were regarded as a testing set to evaluate the predictive capability of the signature. All *P*<0.05 were regarded as a significant difference.

### Gene Set Enrichment Analysis

GSEA was executed to discover changes in the expression of predefined gene sets rather than individual gene, so it can be performed to identify whether gene sets show statistically remarkable differences between the two biological states of samples. In our analysis, GSEA version 4.0.3 was used to verify whether the differentially expressed gene sets between the low-risk group and high-risk group were enriched in cancer-related and autophagy-related processes.

### Cell Culture

Human normal breast cells (HBL-100) and breast cancer cells (MCF-7 and T-47D) were purchased from the Cell Bank of the Chinese Academy of Sciences, Shanghai, China. All cells were incubated in RPMI-1640 medium (Solarbio, Beijing, China) supplemented with 10% fetal bovine serum (Biological Industries, Israel, USA), 100 units/ml penicillin G, and 100 ìg/ml streptomycin. Cells were maintained at 37°C in a damp incubator, which was supplemented with 5% CO2.

### LncRNAs Validation Using qRT-PCR

After being isolated by TRIzol reagent (Invitrogen, Carlsbad, CA, USA) and quantified by NanoDrop 2000 (Thermo Fisher Scientific Inc, Rockford, IL, USA), 1 µg of total RNA underwent reverse transcription by UEIris II RT-PCR System for First-Strand cDNA Synthesis (US EVERBRIGHT INC, Suzhou, China) according to the manufacturer’s instruction. The product was then used for real-time PCR by Universal SYBR Green qPCR Supermix (US EVERBRIGHT INC, Suzhou, China). PCR was conducted as follows: predegeneration at 95°C for 5 min; denaturation at 95°C for 5 sec, annealing at 60°C for 30 sec, repeating the previous process 40 times. GRAPDH was used as internal references, and experiment of each group was repeated three times. Quantification of the relative expression levels of lncRNA by 2^-△△Ct^ method. Primers sequences are presented in **Table 1**.

**Table 1:** Primer sequences for RT-PCR analysis.

	Primer sequences
USP30-AS1-F	5’-TGAAACCGTCTCCTCCGCTACC-3’
USP30-AS1-R	5’-TGTCCTGCGGTCTACGTTCCC-3’
TFAP2A-AS1-F	5’-CTTGACAGCTCCAGGGGTTA-3’
TFAP2A-AS1-R	5’-TCTAGACTTGCAGGCACACA-3’
MAPT-AS1-F	5’-AGATGCACCTGCAGCCC-3’
MAPT-AS1-R	5’-CCCGTCCTTGTTCTGACTCC-3’
LINC01087-F	5’-CCACCAACCTCACCCACTCAAAG-3’
LINC01087-R	5’-TCCTCACGCCTCTGCTCCATC-3’
GAPDH-F	5’-CAGGAGGCATTGCTGATGAT-3’
GAPDH-R	5’-GAAGGCTGGGGCTCATTT-3’

### Statistical Analysis

All statistical analysis and drawings were carried out using R software (version 3.6.3) and Bioconductor. The co-expression network was constructed by Cytoscape to visualize candidate lncRNAs related to prognosis. The autophagy-related lncRNAs in patients with breast cancer were analyzed with R software package “ggalluvial,” “ggplot2,” and “pheatmap.” Then Sankey diagram, risk score, survival status, and lncRNA heat map were drawn. Kaplan Meier-plotter was performed to evaluate the OS differences between high-risk group and low-risk group of the signature. Univariate and multivariate Cox regression were constructed to assess whether the prognostic signature was independent of clinical characteristics such as age, gender, clinical stage, and TNM stage. And the Cox regression also was used to confirm the clinical value of the signature in age, gender, clinical stage, and TNM stage. The above operations are implemented using the R software package “Survival.” The nomogram was implemented using rms R package to predict the OS. All *P*<0.05 was regarded as a significant difference. The Limma package was used to identify the differently expressed lncRNAs in view of |log2 fold change (FC)|≥1 and false discovery rate (FDR)<0.05 based on 9 lncRNAs. All *P*<0.05 were considered statistically significant.

## Results

### Construction of Autophagy-Associated lncRNAs Co-expression Network

**Figure 2:**
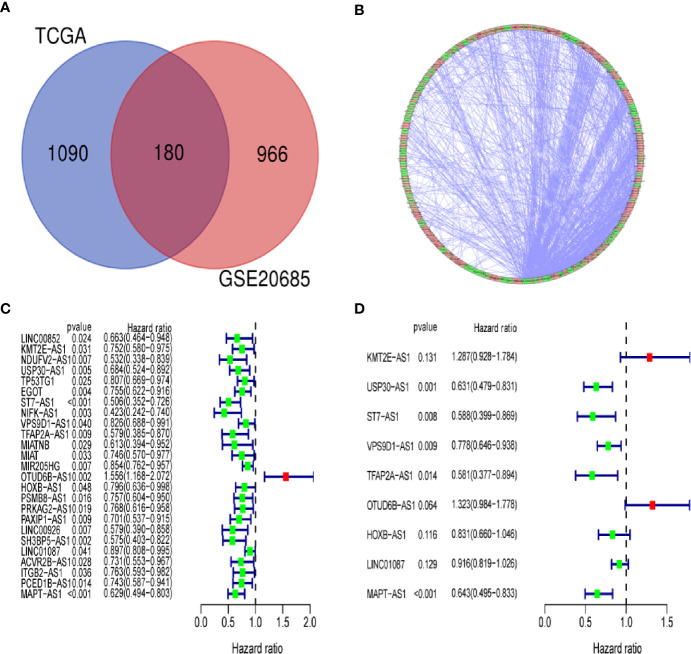
Identification of an autophagy-related lncRNA signature in breast cancer. **(A)** Venn diagram describes 180 shared lncRNAs from 1,270 autophagy-related lncRNAs and 1,146 lncRNAs of GSE20685. **(B)** The network of autophagy-genes and lncRNAs. The blue nodes indicate autophagy genes and the pink nodes indicate lncRNA. The co-expression network is performed by CYTOSCAPE 3.7.2. **(C)** Univariate Cox regression analysis was used to confirm the lncRNAs associated with autophagy were strongly with patients’ OS in training dataset. **(D)** Multivariate Cox regression analysis to construct the prognostic signature.

We identified a total of 19,658 mRNAs and 14,142 lncRNAs, which were screened from the TCGA dataset. According to 222 autophagy genes in Human Autophagy Database, we developed a co-expression network to determine autophagy-related lncRNAs. The Pearson correlation analysis showed that 1,270 lncRNAs were associated with autophagy-related genes in breast cancer (R^2^ > 0.3, *P* < 0.001). Finally, we obtained 180 shared lncRNAs from 1,270 autophagy-related lncRNAs and 1,146 lncRNAs of GSE20685 (**Figures 2A, B**).

### Identification of an Autophagy-Related lncRNA Signature in Breast Cancer

**Figure 3:**
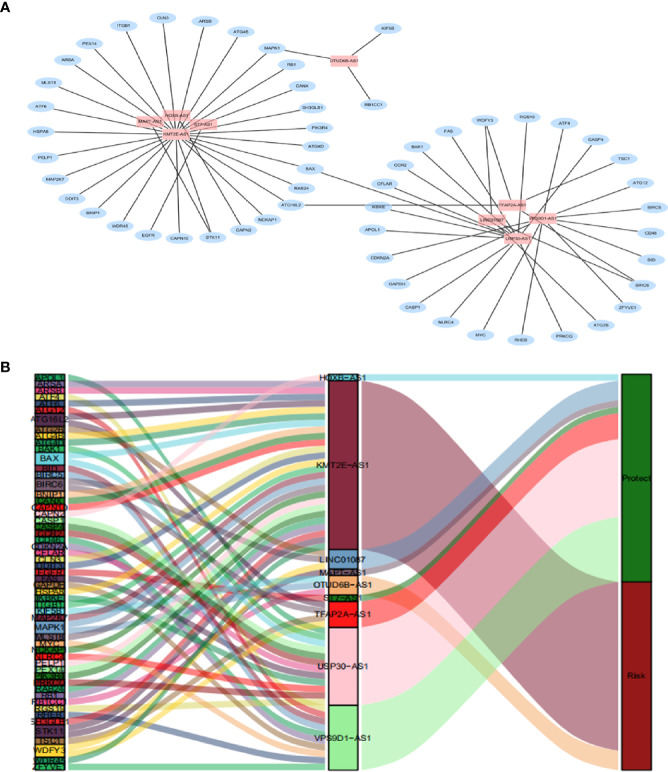
A depiction of the regulation network of the prognostic lncRNAs and autophagy-related genes in breast cancer. **(A)** The blue nodes indicate autophagy genes and the pink nodes indicate lncRNA. The co-expression network is performed by CYTOSCAPE 3.7.2. **(B)** Among the risk types, the lncRNAs linked to dark green is protective lncRNAs, and dark red lncRNAs represents risk lncRNAs.

By using univariate cox regression analysis, we identified 25 lncRNAs that were strongly with patients’ OS from 180 autophagy-related lncRNAs in training dataset (**Figure 2C**) (*P<0.05*). Stepwise multivariable cox proportional hazards regression analysis were carried out to identify the optimal prognostic lncRNAs among the 25 candidate lncRNAs. Subsequently, 9 candidate lncRNAs were identified to be independent prognostic factors for breast cancer patients (**Figure 2D**). We then developed autophagy-related lncRNAs co-expression networks based on the 9 genes (**Table 2**, **Figure 3A**). Next, we constructed a signature of 9 autophagy-related lncRNAs using a risk score method. The prognostic risk score formula of the signature was as follows: prognostic score = (0.2520 × KMT2E-AS1) + (-0.4604 × USP30-AS1) + (-0.5304 × ST7-AS1) + (-0.2505 × VPS9D1) + (-0.5438 × TFAP2A-AS1) + (0.2797 × OTUB6DB-AS1) + (- 0.1847 × HOXB-AS1) + (- 0.0873 ×LINC01087) + (- 0.4423×MAPT-AS1) (*P*<0.05) (**Table 3**). Based on the median risk score, 1,109 patients with breast cancer in training dataset were divided into two groups: high-risk group and low-risk group. In **Figure 3B**, we can see 7 lncRNAs are favorable prognostic factors (USP30-AS1, ST7-AS1, VPS9D1-AS1, TFAP2A-AS1, HOXB-AS1, LINC01087, and MAPT-AS1) and 2 lncRNAs are unfavorable prognostic factors (KMT2E-AS1, OTUB6DB-AS1) for breast cancer patients.

**Table 2:** Correlation between autophagy genes and lncRNAs in breast cancer.

Autophagy genes	lncRNAs	Correlation	*P* value
ARSA	KMT2E-AS1	0.38	7.24E-40
ARSB	KMT2E-AS1	-0.33	2.06E-30
ATF6	KMT2E-AS1	-0.31	2.37E-26
ATG16L2	KMT2E-AS1	0.30	9.31E-25
ATG4B	KMT2E-AS1	0.50	2.12E-72
ATG4D	KMT2E-AS1	0.30	4.97E-25
BAX	KMT2E-AS1	0.46	2.74E-60
BNIP1	KMT2E-AS1	0.31	3.85E-26
CANX	KMT2E-AS1	-0.32	1.44E-27
CAPN10	KMT2E-AS1	0.51	2.75E-75
CLN3	KMT2E-AS1	0.35	5.52E-33
DDIT3	KMT2E-AS1	0.30	1.24E-24
HSPA8	KMT2E-AS1	-0.30	3.95E-25
ITGB1	KMT2E-AS1	-0.43	1.63E-51
MAP2K7	KMT2E-AS1	0.32	2.21E-27
MAPK1	KMT2E-AS1	-0.33	4.61E-29
MLST8	KMT2E-AS1	0.36	2.58E-35
NCKAP1	KMT2E-AS1	-0.40	1.48E-43
PELP1	KMT2E-AS1	0.37	2.15E-36
PEX14	KMT2E-AS1	0.35	8.71E-33
PIK3R4	KMT2E-AS1	-0.33	4.89E-30
RAB24	KMT2E-AS1	0.50	6.73E-72
RB1	KMT2E-AS1	-0.37	1.37E-37
SH3GLB1	KMT2E-AS1	-0.33	8.19E-29
STK11	KMT2E-AS1	0.38	6.93E-39
WDR45	KMT2E-AS1	0.42	6.34E-49
APOL1	USP30-AS1	0.65	2.41E-136
BAK1	USP30-AS1	0.36	3.89E-35
BAX	USP30-AS1	0.30	5.64E-25
CASP1	USP30-AS1	0.63	4.65E-122
CASP4	USP30-AS1	0.51	7.21E-74
CCR2	USP30-AS1	0.51	4.73E-74
CFLAR	USP30-AS1	0.37	2.25E-36
FAS	USP30-AS1	0.42	4.41E-48
IKBKE	USP30-AS1	0.44	7.15E-55
NLRC4	USP30-AS1	0.31	1.82E-26
PRKCQ	USP30-AS1	0.48	6.54E-66
RGS19	USP30-AS1	0.41	7.33E-46
EGFR	ST7-AS1	0.38	4.60E-39
ATF4	VPS9D1-AS1	0.33	8.33E-29
ATG12	VPS9D1-AS1	-0.33	1.00E-28
BID	VPS9D1-AS1	0.37	1.14E-37
BIRC5	VPS9D1-AS1	0.32	1.29E-28
CD46	VPS9D1-AS1	-0.33	5.24E-30
CDKN2A	VPS9D1-AS1	0.33	3.76E-30
GAPDH	VPS9D1-AS1	0.36	7.50E-36
MYC	VPS9D1-AS1	0.45	1.57E-56
RHEB	VPS9D1-AS1	0.32	1.27E-28
ZFYVE1	VPS9D1-AS1	-0.36	4.51E-35
ATG16L2	TFAP2A-AS1	0.34	4.47E-31
BIRC6	TFAP2A-AS1	0.36	2.32E-35
TSC1	TFAP2A-AS1	0.40	5.96E-45
WDFY3	TFAP2A-AS1	0.35	3.17E-34
KIF5B	OTUD6B-AS1	0.31	1.60E-26
MAPK1	OTUD6B-AS1	0.33	5.57E-30
RB1CC1	OTUD6B-AS1	0.59	1.86E-104
CAPN2	HOXB-AS1	0.32	1.41E-28
ATG2B	LINC01087	0.31	2.52E-25
BIRC6	LINC01087	0.34	4.44E-32
WDFY3	LINC01087	0.40	6.65E-44
STK11	MAPT-AS1	0.32	4.11E-27

LncRNAs, long noncoding RNAs; P<0.05 was regarded as a significant difference.

**Table 3:** The 9 autophagy-related lncRNAs significantly associated with OS in the training set.

LncRNAs	β	HR	Lower 95%CI	Upper 95%CI	*P*-value
KMT2E-AS1	0.2520	1.2866	0.9278	1.7840	0.1308
USP30-AS1	-0.4604	0.6310	0.4794	0.8305	0.0010
ST7-AS1	-0.5304	0.5884	0.3985	0.8687	0.0076
VPS9D1-AS1	-0.2505	0.7784	0.6457	0.9383	0.0086
TFAP2A-AS1	-0.5438	0.5805	0.3770	0.8940	0.0136
OTUD6B-AS1	0.2797	1.3227	0.9840	1.7779	0.0638
HOXB-AS1	-0.1847	0.8314	0.6605	1.0464	0.1157
LINC01087	-0.0873	0.9164	0.8188	1.0257	0.1290
MAPT-AS1	-0.4423	0.6426	0.4955	0.8334	0.0009

LncRNAs, long noncoding RNAs; β is the regression coefficient of the corresponding lncRNA; HR, Hazard ratio; P<0.05 was regarded as a significant difference.

### Validation of Prognostic Performance of the Autophagy-Related lncRNA Signature in Breast Cancer

**Figure 4:**
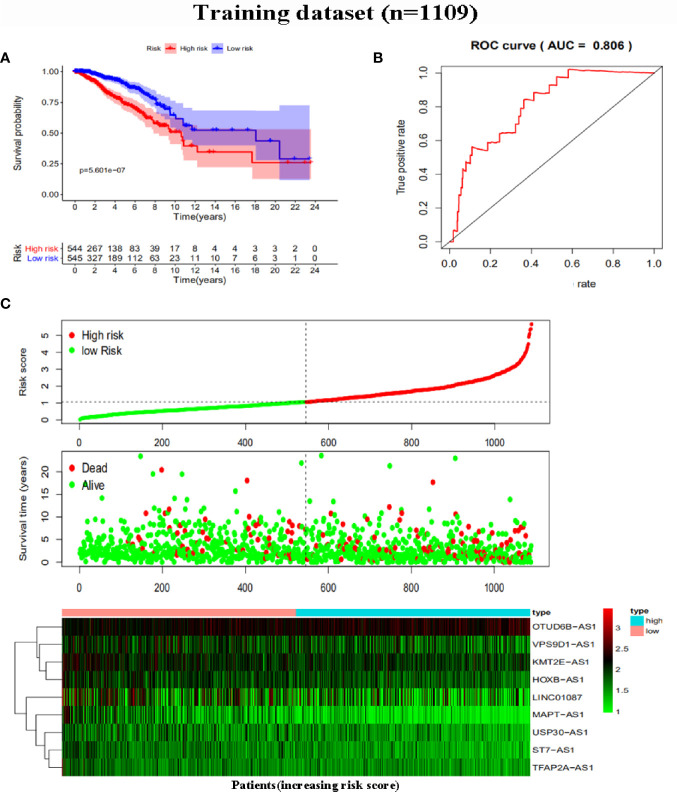
Risk score analysis of the prognostic signature in the training dataset. **(A)** Kaplan-Meier survival analysis for high-risk group and low-risk group. **(B)** ROC curves for predicting OS based on risk score. **(C)** Risk score distribution, survival status, and expression heat map.

**Figure 5:**
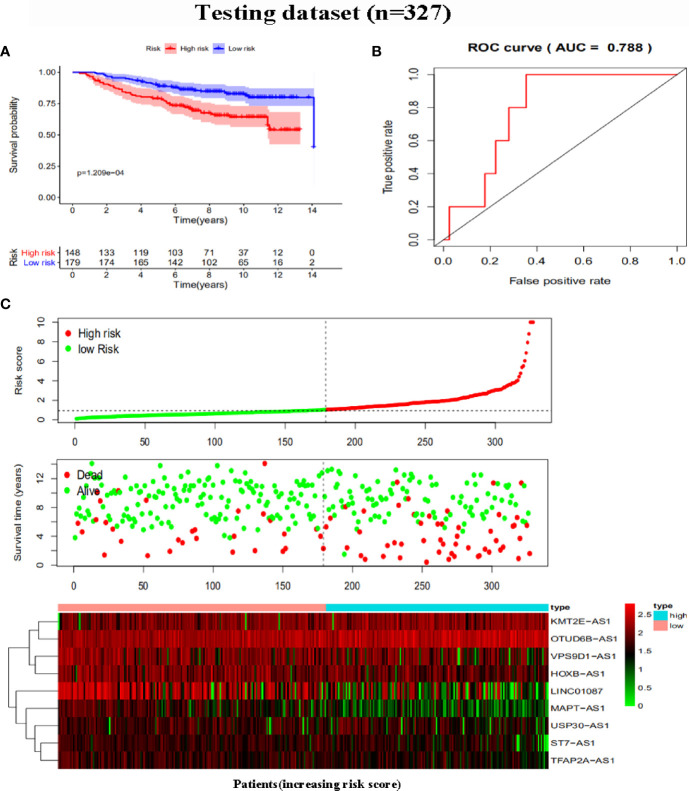
Risk score analysis of the prognostic signature in the testing dataset. **(A)** Kaplan-Meier survival for low-risk group and high-risk group. **(B)** ROC curves for predicting OS based on risk score. **(C)** Risk score distribution, survival status, and expression heat map.

**Figure 6:**
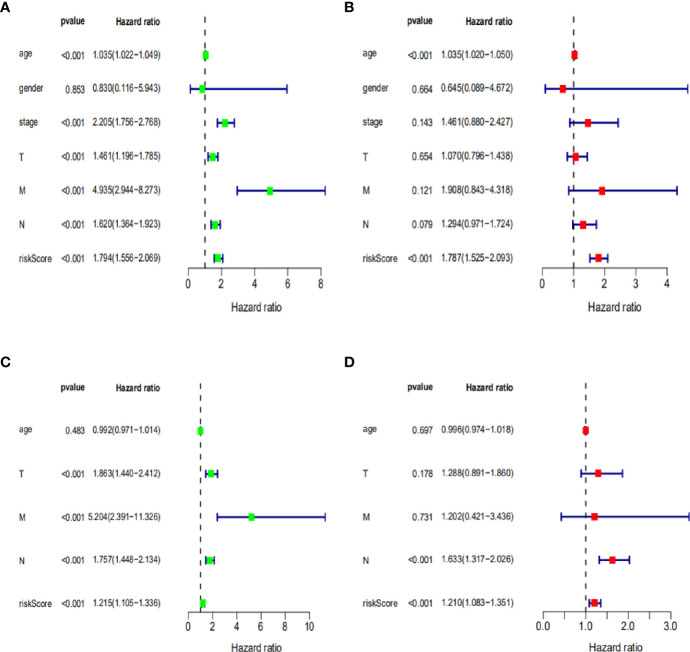
Cox regression analysis of clinical characteristics related to OS in training dataset and testing dataset. **(A, B)** Univariate and multivariate Cox regression analysis of clinical characteristics related to OS in training dataset. **(C, D)** Univariate and multivariate Cox regression analysis of clinical characteristics related to OS in testing dataset.

To further assess the capability of the signature on the prognosis of breast cancer, Kaplan-Meier survival analysis and ROC curve analysis were operated in the training cohort from the TCGA dataset. The results demonstrated that the OS of breast cancer patients in the high-risk group was poorer than the low-risk group based on the prognostic signature (HR: 1.794, 95% CI: 1.556–2.069; *P*<0.001) (**Figure 4A**). Additionally, **Figure 4B** illustrated that risk score of the signature had an AUC of 0.806 under the ROC curve, indicating a credible diagnostic value. The risk score of the prognostic signature in the low-risk group and high-risk group, survival status of breast cancer patients, and the expression heat map are displayed in **Figure 4C**. To assess the robustness of the signature in OS prediction of breast cancer patients, we further examined it in the testing cohort from the GSE20685 dataset using the same formula. The results revealed that the OS of patients with high-risk score was also worse than those with low-risk score in the GSE20685 cohort (HR: 1.215, 95% CI: 1.105–1.336; *P*<0.001) and the AUC of the ROC curve of the prognostic model was 0.788 (**Figures 5A–C**). This result is consistent with the training set. Finally, Cox regression analyses were used to confirm the possibility that risk score of the signature can be regarded as an independent predictor of prognosis in breast cancer patients. The results indicated that whether in the training dataset or the testing dataset, the risk score of the signature could significantly help to forecast the prognosis of breast cancer patients, eliminating the influence of clinical features (gender, age, clinical stage, and TNM stage) (*P*<0.001) (**Table 4**, **Figures 6A–D**).

**Table 4:** Predictive values of related clinical features and risk score in the training dataset and the testing dataset.

Variables	Univariate analysis			Multivariate analysis		
	HR	95% CI	*P-*value	HR	95% CI	*P-*value
Training dataset						
age	1.035	1.022–1.049	<0.001	1.035	1.020–1.050	<0.001
gender	0.830	0.116–5.943	0.853	0.645	0.089–4.672	0.664
stage	2.205	1.756–2.768	<0.001	1.461	0.880–2.427	0.143
T	1.461	1.196–1.785	<0.001	1.070	0.796–1.438	0.654
M	4.935	2.944–8.273	<0.001	1.908	0.843–4.318	0.121
N	1.620	1.364–1.923	<0.001	1.294	0.971–1.724	0.079
risk score	1.794	1.556–2.069	<0.001	1.787	1.525–2.093	<0.001
Testing dataset						
age	0.992	0.971–1.014	0.483	0.996	0.974–1.018	0.697
T	1.863	1.440–2.412	<0.001	1.288	0.891–1.860	0.178
M	5.204	2.391–11.326	<0.001	1.202	0.421–3.436	0.731
N	1.757	1.448–2.134	<0.001	1.633	1.317–2.026	<0.001
risk score	1.215	1.105–1.336	<0.001	1.210	1.083–1.351	0.001

HR, hazard ratio; P<0.05 was regarded as a significant difference.

### Clinical Value of the Prognostic Signature in Patients With Breast Cancer

We further evaluated the clinical value of the signature in breast cancer patients by determining the correlation between lncRNA and the clinical characteristics (age, gender, clinical stage, and TNM stage) of breast cancer patients. The results showed that the risk score of patients over 65 years old (*t*= -2.673, *P*=0.003), stage III–IV (*t*= -2.433, *P*=0.000) and N1–3 (*t*= -1.799, *P*=0.013) tends to increase significantly, suggesting that older age, advanced stage, and lymphatic metastasis may be correlated with the progression of breast cancer (**Table 5**).

**Table 5:** Clinical impact of risk score signature for the TCGA cohort.

Clinical Features	n	Risk Score			
		Mean	SD	t	*P*
Age					
<=65 >65	665	1.270	0.857	-2.673	0.008
	243	1.452	0.932		
Gender					
Female Male	897	1.319	0.882	0.314	0.700
	11	1.238	0.853		
Stage					
I-II III-IV	691	1.277	0.862	-2.443	0.000
	217	1.451	0.929		
T					
T1-2 T3-4	774	1.304	0.870	-1.169	0.161
	134	1.405	0.938		
M					
M0 M1	891	1.310	0.881	-2.325	0.186
	17	1.746	0.763		
N					
N0 N1-3	449	1.265	0.867	-1.799	0.013
	459	1.370	0.892		

P<0.05 was regarded as a significant difference.

### Gene Set Enrichment Analysis

**Figure 7:**
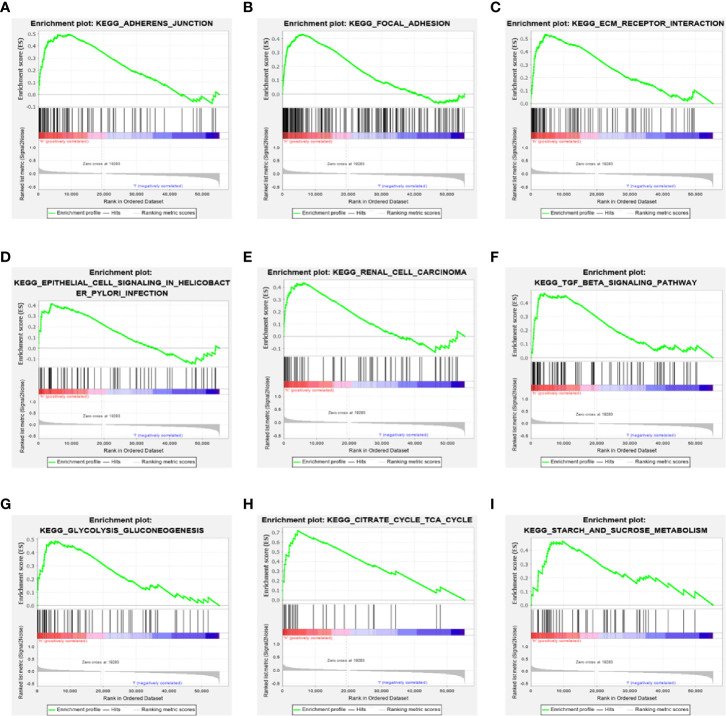
Gene set enrichment analysis. **(A–F)** GSEA suggested notably enrichment of cancer-related pathways in the high-risk group based on training set. **(G–I)** GSEA suggested significant autophagy-related enrichment based on training set.

To further explore the potential biological behavior of this signature in breast cancer patients, we determined a total of 25 gene sets using GSEA, which were notably enriched with a nominal *P* value of < 0.05 and FDR *q*-value < 0.25 (**Table 6**). The results revealed that the differentially expressed genes between the high-risk and low-risk groups were enriched in the cancer-related and autophagy-related pathways. As shown in **Table 5** and **Figures 7A–F**, stromal and carcinogenic activation pathways were markedly enriched in high risk group, such as cell adhesion, ECM receptor interaction, TGF beta signaling pathway, Renal cell carcinoma pathway, and Prostate cancer pathway. Surprisingly, we also found that glucose metabolic pathways were significant such as Glycolysis/Gluconeogenesis pathway, TCA cycle pathway, and starch and sucrose metabolism pathway enriched in high risk groups (**Figures 7G–I**). It is worth noting that TGF-β signaling pathway and glucose metabolism pathway are closely related to autophagy. The results from GSEA have revealed these autophagy-related genes contribute to carcinogenic activation pathways and autophagy-related pathways, which may provide sufficient evidence for targeted therapy of breast cancer.

**Table 6:** Gene set enrichment analysis based on the signature of 9 autophagy-related lncRNAs.

Name	Size	ES	NES	NOM *P*-val	FDR*q*-val	FWER*P*-val	Rank at max	Leading edge
oocyte_meiosis	112.000	0.536	2.039	0.000	0.047	0.066	4649.000	tags=39%, list=8%, signal=43%
vibrio_cholerae_infection	54.000	0.570	1.956	0.002	0.087	0.150	8855.000	tags=52%, list=16%, signal=62%
gap_junction	90.000	0.449	1.760	0.002	0.138	0.414	6344.000	tags=39%, list=11%, signal=44%
citrate_cycle_tca_cycle	31.000	0.718	2.040	0.006	0.095	0.066	4597.000	tags=55%, list=8%, signal=60%
pathogenic_escherichia_coli_infection	56.000	0.546	1.789	0.006	0.217	0.363	6053.000	tags=43%, list=11%, signal=48%
o_glycan_biosynthesis	30.000	0.534	1.758	0.010	0.127	0.417	7284.000	tags=50%, list=13%, signal=58%
steroid_biosynthesis	17.000	0.700	1.776	0.011	0.151	0.384	2995.000	tags=53%, list=5%, signal=56%
tgf_beta_signaling_pathway	85.000	0.469	1.752	0.012	0.112	0.427	3973.000	tags=31%, list=7%, signal=33%
glycolysis_gluconeogenesis	62.000	0.488	1.771	0.013	0.140	0.396	5471.000	tags=34%, list=10%, signal=38%
terpenoid_backbone_biosynthesis	15.000	0.705	1.786	0.015	0.186	0.368	5030.000	tags=40%, list=9%, signal=44%
n_glycan_biosynthesis	46.000	0.548	1.783	0.016	0.163	0.371	4408.000	tags=39%, list=8%, signal=42%
regulation_of_actin_cytoskeleton	213.000	0.401	1.643	0.016	0.161	0.576	6127.000	tags=35%, list=11%, signal=39%
adherens_junction	73.000	0.497	1.836	0.017	0.192	0.291	9394.000	tags=52%, list=17%, signal=63%
renal_cell_carcinoma	70.000	0.435	1.643	0.023	0.170	0.575	5850.000	tags=37%, list=11%, signal=41%
progesterone_mediated_oocyte_maturation	85.000	0.434	1.621	0.026	0.173	0.611	4505.000	tags=33%, list=8%, signal=36%
starch_and_sucrose_metabolism	52.000	0.468	1.568	0.028	0.202	0.684	9541.000	tags=44%, list=17%, signal=53%
protein_export	24.000	0.643	1.687	0.029	0.137	0.514	4379.000	tags=50%, list=8%, signal=54%
dorso_ventral_axis_formation	24.000	0.549	1.717	0.029	0.122	0.473	5696.000	tags=42%, list=10%, signal=46%
epithelial_cell_signaling_in_helicobacter_pylori_infection	68.000	0.416	1.538	0.035	0.209	0.728	3970.000	tags=29%, list=7%, signal=32%
arrhythmogenic_right_ventricular_cardiomyopathy_arvc	74.000	0.450	1.596	0.038	0.180	0.651	6112.000	tags=35%, list=11%, signal=39%
long_term_potentiation	70.000	0.387	1.557	0.040	0.197	0.697	6344.000	tags=31%, list=11%, signal=35%
ecm_receptor_interaction	84.000	0.536	1.726	0.040	0.122	0.454	4669.000	tags=36%, list=8%, signal=39%
focal_adhesion	199.000	0.433	1.610	0.046	0.175	0.628	6112.000	tags=36%, list=11%, signal=41%
prostate_cancer	89.000	0.405	1.534	0.046	0.206	0.730	4558.000	tags=33%, list=8%, signal=35%
amino_sugar_and_nucleotide_sugar_metabolism	43	0.476	1.568	0.047	0.194	0.684	5323	tags=37%, list=10%, signal=41%

ES, enrichment score; NOM P-value, nominal P-value; FDR, false discovery rate; FWER, familywise-error rate; NES, normalized enrichment score.

### The Nomogram Establishing and the Prognostic Value Validating Base on 9 lncRNAs in TCGA Dataset

**Figure 8:**
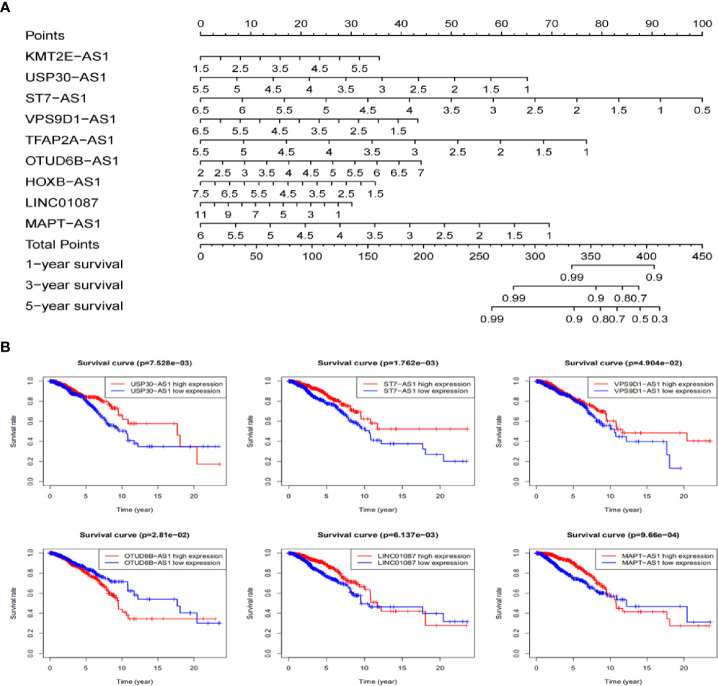
The nomogram establishing and the prognostic value validating base on 9 lncRNAs in TCGA dataset. **(A)** Establishment of a nomogram for 1-, 3-, and 5-year OS prediction in breast cancer. **(B)** Validation the prognostic value of these 9 autophagy-related lncRNAs in breast cancer by Kaplan Meier-plotter curve.

Based on these autophagy-related lncRNAs of the signature, a nomogram was constructed. Multivariate Cox analysis was performed to assign the points in the nomogram to each variable. By drawing a vertical line between the total point axis and each prognostic axis, the estimated survival rate of breast cancer patients at 1, 3, and 5 years can be calculated, which may help professionals make clinical decisions for breast cancer patients (**Figure 8A**). In order to further explore the prognostic value of the 9 lncRNAs, the Kaplan Meier curve was built to confirm the relationship between these lncRNAs and OS. In our analysis, a total of 6 of the 9 lncRNAs (USP30-AS1, ST7-AS1, VPS9D1-AS1, OTUB6DB-AS1, LINC01087, and MAPT-AS1) were identified. The results indicated that the 6 autophagy-related lncRNAs were correlated to the OS in breast cancer patients (**Figure 8B**).

### Validation the Expression of 9 lncRNAs in Breast Cancer Tissues and Cells

**Figure 9:**
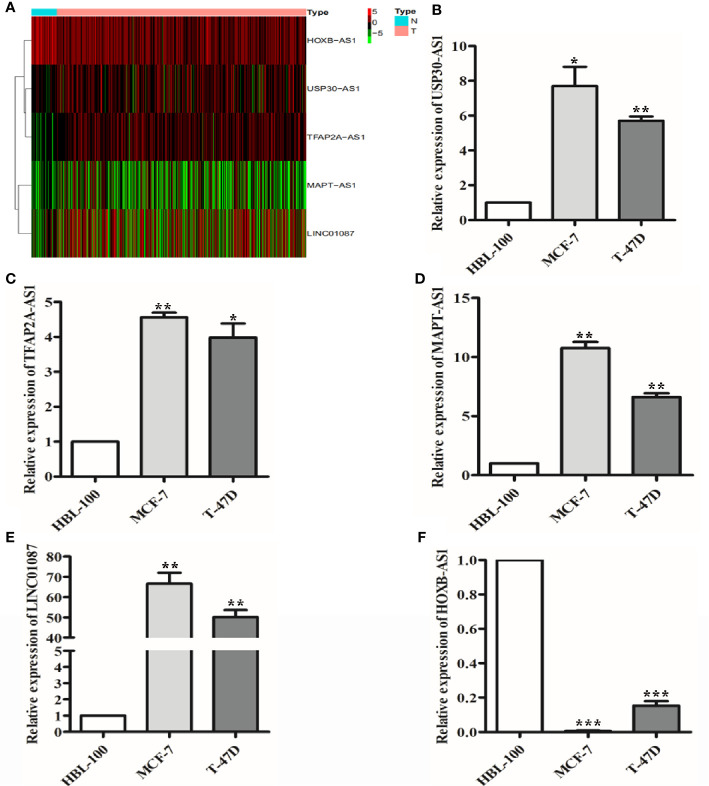
The expression of 9 lncRNAs in breast cancer tissues and cells. **(A)** Heat map of 5 lncRNAs with significantly differential expression in breast cancer tissues. **(B–E)** qRT-PCR results showed that USP30-AS1, TFAP2A-AS1, MAPT-AS1, and LINC01087 expression were higher in breast cancer cell lines than in the normal cell lines. *P<0.05, **P<0.01 **(F)** qRT-PCR results showed that HOXB-AS1 expression was lower in breast cancer cell lines than in the normal cell lines. ***P<0.001.

To further validate the expression profiles of the 9 lncRNAs, we explored the expression levels of the lncRNAs in breast cancer tissues based on TCGA database as well as in breast cancer lines by qRT-PCR. The results showed that USP30-AS1, TFAP2A-AS1, MAPT-AS1, and LINC01087 expression were significantly increased and HOXB-AS1 expression was significantly decreased in breast cancer tissues compared with normal breast tissue (**Figure 9A**, *P*<0.001). qRT-PCR results showed that USP30-AS1, TFAP2A-AS1, MAPT-AS1, and LINC01087 were overexpressed and HOXB-AS1 was lowexpressed in MCF-7 and T-47D cell lines compared to HBL-100 cell line (**Figures 9B–F**, *P*<0.05).

## Discussion

Breast cancer is characterized by the accumulation of abnormal cells, which may be attributed to the imbalance of cell proliferation, apoptosis, and autophagy regulation disorder. Autophagy plays a dual role in inhibiting and promoting the occurrence and development of cancer at different stages (24). Increasing evidence shows that lncRNAs and autophagy are closely associated with the occurrence, development, and prognosis of many different tumors. Recent evidence suggests that lncRNA NAMPT-AS promoted breast cancer progression and regulated autophagy through the mTOR pathway (25). Existing studies showed that there is a positive relationship between the expression of lncRNA LCPAT1 and LC3β in lung cancer (26). Other studies showed that lncRNA DICER1-AS1 expression was up-regulated in osteosarcoma cells, while knockdown of DICER1-AS1 could inhibit the proliferation, migration, and invasion of osteosarcoma cells, and reduce the protein expression levels of ATG5, LC3, and Beclin1, indicating that knocking down DICER1-AS1 can inhibit autophagy of osteosarcoma cells (27).

The specific mechanisms of lncRNAs regulating autophagy can be divided into three categories: lncRNAs act as competitive endogenous RNAs (ceRNAs) combined with miRNAs to regulate the expression of miRNAs, thus affecting the process of autophagy (28); lncRNAs can also affect the expression of ATG gene cis or trans (24); lncRNAs promote tumor progression by inhibiting autophagy-mediated apoptosis through AKT/mTOR pathway (29). LncRNAs regulate the occurrence and development of autophagy in both directions, which provides new ideas and ways for further research on the prevention and treatment of autophagy-related diseases, such as cancer. At present, using high-throughput biotechnology to detect genetic changes to predict tumor recurrence and metastasis has become a research hotspot. However, a single lncRNA may not be sufficient to forecast the prognosis of the breast cancer patients. Therefore, it is urgent to construct an autophagy-related lncRNA signature to predict the survival of breast cancer patients.

In our analysis, the TCGA dataset and GSE20685 dataset were downloaded to investigate the prognosis signature of autophagy-related lncRNAs for breast cancer patients. Firstly, we identified 1,270 lncRNAs associated with autophagy-related genes in breast cancer (R^2^ > 0.3, *P* < 0.001). Because the breast cancer samples in TCGA and GEO databases are different, the extracted lncRNAs are not exactly the same. We analyzed the common lncRNAs in the two databases to ensure that these lncRNAs are general and universal in breast cancer samples, and narrow the scope of biological marker screening. Then we obtained 180 shared lncRNAs from TCGA dataset and GSE20685 dataset. We further identified a signature composed of 9 autophagy-related lncRNAs that could divide breast cancer patients into high-risk group or low-risk group. Among the 9 autophagy-related lncRNAs, USP30-AS1, ST7-AS1, VPS9D1-AS1, TFAP2A-AS1, HOXB-AS1, LINC01087, and MAPT-AS1 are protective factors, while KMT2E-AS1 and OTUB6DB-AS1 are risk-related factors. In addition, it was also found that the OS of breast cancer patients in the high-risk group was poorer than that in the low-risk group based on the prognostic signature. The validation in training set and testing set revealed that the prognostic signature has better diagnostic capability. Besides, we used Cox regression analysis that concluded that the signature is an independent factor of breast cancer. We also evaluated the clinical value of the signature in age, gender, clinical stage, and TNM stage and found that the signature may be associated with the progression of breast cancer. To further analyze these 9 lncRNAs of the signature, we developed a nomogram and validated their prognostic value and expression. We found USP30-AS1, ST7-AS1, VPS9D1-AS1, OTUB6DB-AS1, LINC01087, and MAPT-AS1 were correlated to the OS in breast cancer patients. And we further verified that USP30-AS1, TFAP2A-AS1, MAPT-AS1, and LINC01087 were significantly increased and HOXB-AS1 was significantly decreased in breast cancer tissues and cells.

Moreover, in our analysis, we found that several pathways have been established in cancer such as ECM receptor interaction, TGF beta signaling pathway, cell adhesion, Renal cell carcinoma pathway, and Prostate cancer pathway, which were markedly enriched in high risk group. We also detect that the glucose metabolism pathways were enriched in high-risk patients, especially glycolysis, gluconeogenesis, and tricarboxylic acid cycle. Previous studies have shown that abnormal cell metabolism, especially glucose metabolism, is related to the occurrence and progression of tumors (30, 31). Tumor cells absorb a large amount of glucose and tend to carry out glycolysis in the cytoplasm even under the condition of sufficient oxygen supply, thus promoting the rapid growth and proliferation of cells (31, 32). Recent studies have found an unexpected link between glucose metabolism and autophagy. They found that increase of glycolysis in autophagic cells can promote Ras-mediated adhesion-independent transformation, indicating autophagy may promote Ras-driven tumor growth in a specific metabolic environment (33). This is consistent with our present findings.

However, the molecular mechanism of these 9 autophagy-related lncRNAs is still inadequately understood in breast cancer, and further investigation of the underlying mechanisms may be meaningful. So we established a nomogram to more intuitively predict the OS in 1, 3, and 5 years. Subsequently, we discover the prognostic value of these 9 autophagy-related lncRNAs using the Kaplan-Meier survival analysis and the results were basically consistent with the prognostic analysis results of TCGA dataset. Furthermore, targeted analysis on a specific histological group of breast cancer patients will increase the specificity and individuality of the autophagy-related lncRNAs. In our further study, we will analyze and verify these lncRNAs in breast cancer patients with the specific morphological and molecular features.

Taken together, our study defines an innovative autophagy-related lncRNA signature in breast cancer. It is a comprehensive analysis of RNA sequencing data and clinical information available in TCGA database. The autophagy-related lncRNA signature may provide new insights into predicting the prognosis of patients with breast cancer. More importantly, the functions and approaches associated with our signature may contribute to the development of a new therapeutic strategy for breast cancer.

## Data Availability Statement

Publicly available datasets were analyzed in this study. This data can be found here: https://www.cancergenome.nih.gov/, https://www.ncbi.nlm.nih.gov/geo/query/acc.cgi?acc=GSE20685, http://www.autophagy.lu/.

## Author Contributions

This study was designed by YZ, JY, and RZ. Data were analyzed by RZ, QZ, DY, ZY, JG, and JZ. The original manuscript was written by RZ. Supervision and manuscript revision were performed by YZ and JY. Funding was acquired by YZ and JY. All authors contributed to the article and approved the submitted version.

## Funding

This work was supported by the National Natural Science Foundation of China (No.: 81402266, 81572747, 52111005), the key research project of Henan high school (18A320051), the Medical Science and Technology Research Projects of Henan Province (No.: SBGJ202002082), and Henan Scientific and Technological Research Projects (No.: 202102310044, 162102410057). Support has also been provided by the Outstanding Young Talent project of Scientific and Technological Innovation in Henan Health (No.: YXKC2020032) and the Clinical Pharmacy Branch of Chinese Medical Association-Wu Jieping Medical Foundation (No.: 320.6750.19090-3).

## Conflict of Interest

The authors declare that the research was conducted in the absence of any commercial or financial relationships that could be construed as a potential conflict of interest.
